# Prevalence, Causes, and Health Care Burden of Pleural Effusions Among Hospitalized Adults in China

**DOI:** 10.1001/jamanetworkopen.2021.20306

**Published:** 2021-08-10

**Authors:** Panwen Tian, Rong Qiu, Meifang Wang, Shufeng Xu, Liming Cao, Ping Yang, Weimin Li

**Affiliations:** 1 Department of Respiratory and Critical Care Medicine, Lung Cancer Treatment Center, West China Hospital, Sichuan University, Chengdu, China; 2 Department of Respiratory and Critical Care Medicine, Suining Central Hospital, Suining, China; 3 Department of Respiratory and Critical Care Medicine, Affiliated Taihe Hospital of Hubei University of Medicine, Shiyan, China; 4 Department of Respiratory and Critical Care Medicine, First Hospital of Qinhuangdao, Qinhuangdao, China; 5 Department of Respiratory and Critical Care Medicine, Xiangya Hospital Central South University, Changsha, China; 6 Division of Epidemiology, Department of Health Sciences Research, Mayo Clinic, Scottsdale, Arizona; 7 Department of Respiratory and Critical Care Medicine, West China Hospital, Sichuan University, Chengdu, China

## Abstract

This cross-sectional study investigates the estimated prevalence, common causes, and health care burden of pleural effusions among hospitalized adults in China.

## Introduction

More than 50 causes of pleural effusion (PE) are recognized.^1^ An estimated 1.5 million patients in the US experience PE each year, with most cases caused by congestive heart failure, pneumonia, and cancer.^2,3^ In 2012, 126 800 people were hospitalized for PE at a cost of more than $5 billion in the US.^3^ However, studies on the epidemiology of PE in the Chinese population are scant. We investigated the prevalence, causes, and health care burden of PE in China.

## Methods

We conducted a multicenter, cross-sectional study including a nationally representative sample of patients at 56 general hospitals from 50 municipalities in mainland China. All tertiary hospitals in each region of China were sampled. The ratio of included hospitals in each region’s capital and noncapital cities was 1:1.

Discharge records of adults who had received a diagnosis of PE and were admitted to the inpatient departments between January 1 and December 31, 2018, were reviewed. Patients with PE confirmed by ultrasonography or computed tomography (CT) were included. A panel of experts confirmed the diagnosis of PE and determined its etiology according to international guidelines (see eAppendix in the Supplement). Race/ethnicity data were not collected. The Ethics Committee of West China Hospital, Sichuan University reviewed and approved this study and provided a waiver of informed consent because of the retrospective nature of this study. This study followed the Strengthening the Reporting of Observational Studies in Epidemiology (STROBE) reporting guideline.

The χ^2^ test was used to compare the differences in causes of PE among patients with different smoking statuses. Data were analyzed with SPSS version 21.0 (IBM Corp) from July to September 2020. Statistical significance was defined as 2-sided *P* < .05.

## Results

In total, 24 711 eligible patients were included. Among them, 15 540 (62.9%) were male patients; the mean (SD) age was 61.6 (16.9) years. The estimated prevalence of PE in our sample was 4684 per 1 million Chinese adults (95% CI, 4675-4692 per 1 million Chinese adults). The 3 most common causes were parapneumonic pleural effusion and empyema (6210 patients [25.1%]), malignant neoplasm (5849 patients [23.7%]), and tuberculosis (3035 patients [12.3%]). Tuberculosis was the most common cause in patients aged 18 to 39 years (1063 patients [4.3%]); malignant neoplasm was the most common cause in patients aged 60 to 79 years (3218 patients [13.0%]); and parapneumonic pleural effusion and empyema was the most common cause in patients aged 40 to 59 years (1756 patients [7.1%]) and those aged 80 years and older (1354 patients [5.5%]) (Table). Smokers were more likely than nonsmokers to have malignant PE (33.1% vs 28.3%; *P* < .001; χ^2^_1_ = 45.3) and tuberculous PE (15.7% vs 14.9%; *P* = .03; χ^2^_1_ = 4.6) (Figure). The median hospitalization cost was ¥15 534.5 (interquartile range, ¥9447.2-¥29 000.0) (US $2401.4 [interquartile range, US $1460.4-$4483.1]).

**Table.** Causes of Pleural Effusions in Hospitalized Chinese Patients, by Age Group

Causes of pleural effusion	Patients, No. (%)				
	Total	Age, y			
		18-39	40-59	60-79	≥80
Total	24 711 (100.0)	2860 (11.6)	7008 (28.4)	11 294 (45.7)	3549 (14.4)
PPPE and empyema					
Total	6210 (25.1)	601 (2.4)	1756 (7.1)	2499 (10.1)	1354 (5.5)
Single cause	3939 (15.9)	421 (1.7)	1173 (4.7)	1574 (6.4)	771 (3.1)
Compound causes	2271 (9.2)	180 (0.7)	583 (2.4)	925 (3.7)	583 (2.4)
Malignant neoplasm					
Total	5849 (23.7)	276 (1.1)	1730 (7.0)	3218 (13.0)	625 (2.5)
Single cause	4223 (17.1)	157 (0.6)	1297 (5.2)	2391 (9.7)	378 (1.5)
Compound causes	1626 (6.6)	119 (0.5)	433 (1.8)	827 (3.3)	247 (1.0)
Tuberculosis					
Total	3035 (12.3)	1063 (4.3)	862 (3.5)	945 (3.8)	165 (0.7)
Single cause	2356 (9.5)	898 (3.6)	665 (2.7)	690 (2.8)	103 (0.4)
Compound causes	679 (2.7)	165 (0.7)	197 (0.8)	255 (1.0)	62 (0.3)
Congestive heart failure					
Total	2036 (8.2)	60 (0.2)	309 (1.3)	1115 (4.5)	552 (2.2)
Single cause	1707 (6.9)	53 (0.2)	254 (1.0)	938 (3.8)	462 (1.9)
Compound causes	329 (1.3)	7 (0)	55 (0.2)	177 (0.7)	90 (0.4)
Hypoproteinemia					
Total	759 (3.1)	66 (0.3)	199 (0.8)	362 (1.5)	132 (0.5)
Single cause	747 (3.0)	66 (0.3)	196 (0.8)	356 (1.4)	129 (0.5)
Compound causes	12 (0)	0	3 (0)	6 (0)	3 (0)
Cirrhosis					
Total	672 (2.7)	47 (0.2)	303 (1.2)	282 (1.1)	40 (0.2)
Single cause	465 (1.9)	35 (0.1)	207 (0.8)	195 (0.8)	28 (0.1)
Compound causes	207 (0.8)	12 (0)	96 (0.4)	87 (0.4)	12 (0)
Injury					
Total	385 (1.6)	71 (0.3)	177 (0.7)	124 (0.5)	13 (0.1)
Single cause	373 (1.5)	71 (0.3)	169 (0.7)	121 (0.5)	12 (0)
Compound causes	12 (0)	0	8 (0)	3 (0)	1 (0)
Pericardial disease					
Total	280 (1.1)	17 (0.1)	59 (0.2)	138 (0.6)	66 (0.3)
Single cause	185 (0.7)	13 (0.1)	43 (0.2)	92 (0.4)	37 (0.1)
Compound causes	95 (0.4)	4 (0)	16 (0.1)	46 (0.2)	29 (0.1)
Nephrotic syndrome					
Total	275 (1.1)	69 (0.3)	76 (0.3)	104 (0.4)	26 (0.1)
Single cause	156 (0.6)	36 (0.1)	44 (0.2)	62 (0.3)	14 (0.1)
Compound causes	119 (0.5)	33 (0.1)	32 (0.1)	42 (0.2)	12 (0)
Connective tissue disease					
Total	215 (0.9)	76 (0.3)	71 (0.3)	63 (0.3)	5 (0)
Single cause	134 (0.5)	42 (0.2)	44 (0.2)	47 (0.2)	1 (0)
Compound causes	81 (0.3)	34 (0.1)	27 (0.1)	16 (0.1)	4 (0)
Abdominal disease					
Total	180 (0.7)	30 (0.1)	72 (0.3)	59 (0.2)	19 (0.1)
Single cause	147 (0.6)	30 (0.1)	61 (0.2)	48 (0.2)	8 (0)
Compound causes	33 (0.1)	0	11 (0)	11 (0)	11 (0)
Unknown cause	4026 (16.3)	400 (1.6)	1220 (4.9)	1901 (7.7)	505 (2.0)
Others^a^	789 (3.2)	84 (0.3)	174 (0.7)	484 (2.0)	47 (0.2)

Abbreviation: PPPE, parapneumonic pleural effusion.

^a^ Others include glomerulonephritis, medicine, hemothorax, surgery, obstruction of superior vena cava, chylothorax, uremia, sarcoidosis, diseases of obstetrics and gynecology, pulmonary embolism, and pulmonary hypertension.

**Figure.**
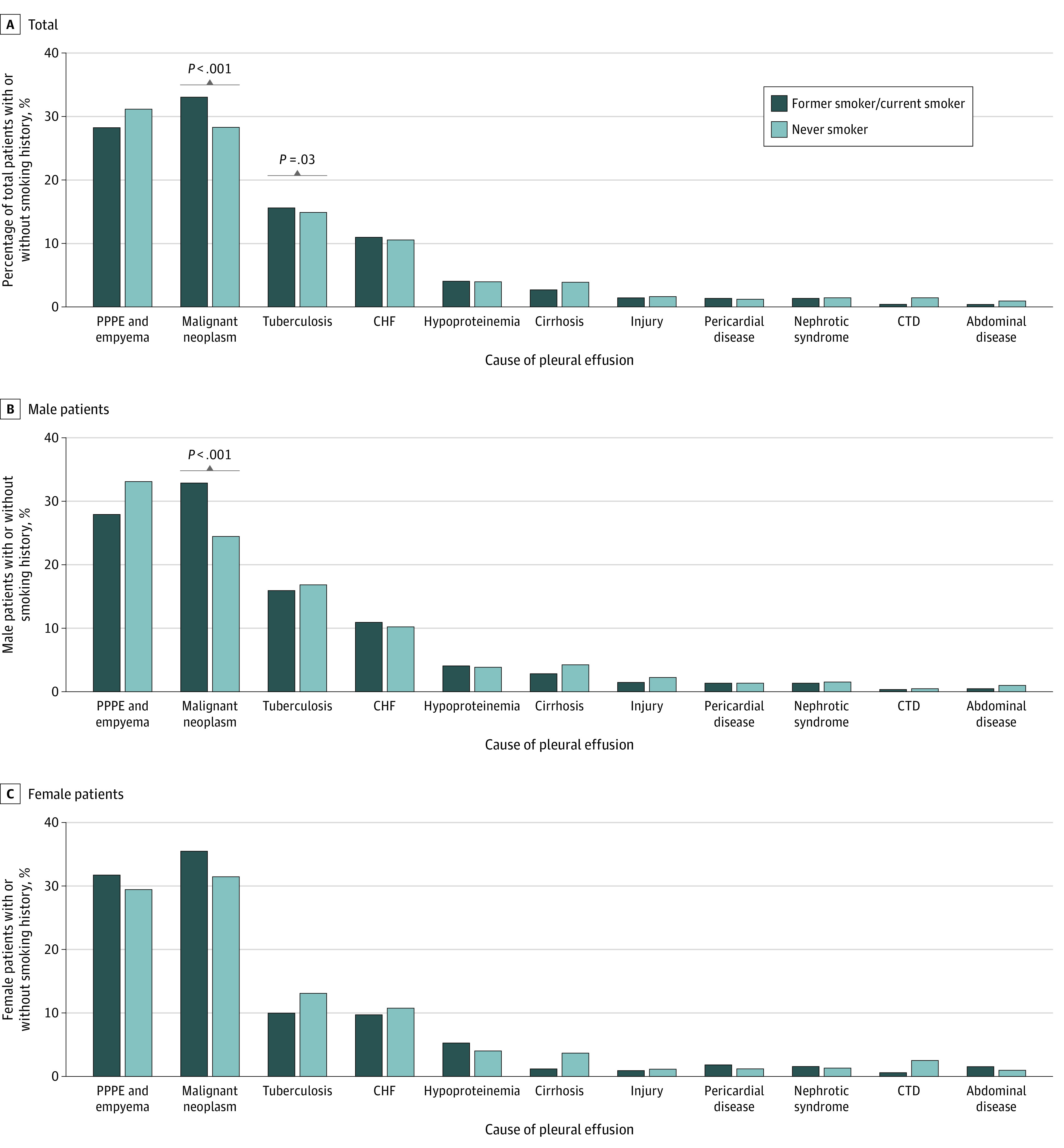
Pleural Effusions Among Patients With or Without a Smoking History CHF indicates congestive heart failure; CTD, connective tissue disease; PPPE, parapneumonic pleural effusion.

## Discussion

This study found that the number of patients with PE in China is high. Although causes of PE have also been reported,^2,4^ we found that the causes differed by age and smoking status. Total hospitalization costs per patient with PE are similar to those per patient with chronic obstructive pulmonary disease (US $1964 to $3449 per patient), representing 33% to 40% of the mean household income in China, increasing the total cost of inpatient care for both individual and social health insurance.^5^

A limitation of this study was its retrospective design; there was potential selection bias. Patients who were clinically suspected of having PE but with no confirmation by CT or ultrasound were excluded, which led to an underestimation of the disease burden.

To our knowledge, this study provided the largest Chinese data set on the prevalence, causes, and health care burden of PE. Policy makers and health care professionals should address this concern by considering age and smoking factors when developing preventions and treatments for patients with PE.

## Notes

Supplement. **eAppendix.** Supplementary Methods
eReferences

## References

[1] Bhatnagar R, Maskell N. The modern diagnosis and management of pleural effusions. BMJ. 2015;351:h4520. doi:10.1136/bmj.h4520 26350935

[2] Light RW. Pleural effusions. Med Clin North Am. 2011;95(6):1055-1070. doi:10.1016/j.mcna.2011.08.005 22032427

[3] Taghizadeh N, Fortin M, Tremblay A. US hospitalizations for malignant pleural effusions: data from the 2012 national inpatient sample. Chest. 2017;151(4):845-854. doi:10.1016/j.chest.2016.11.010 27876589

[4] Puchalski JT, Argento AC, Murphy TE, . Etiologies of bilateral pleural effusions. Respir Med. 2013;107(2):284-291. doi:10.1016/j.rmed.2012.10.004 23219348 PMC5421548

[5] Srivastava K, Thakur D, Sharma S, Punekar YS. Systematic review of humanistic and economic burden of symptomatic chronic obstructive pulmonary disease. Pharmacoeconomics. 2015;33(5):467-488. doi:10.1007/s40273-015-0252-4 25663178

