# Supplementary material for: Prevalence, Causes, and Health Care Burden of Pleural Effusions Among Hospitalized Adults in China
Source: JAMA Netw Open. 2021 Aug 10;4(8):e2120306. doi: 10.1001/jamanetworkopen.2021.20306 (PMC8356070; doi:10.1001/jamanetworkopen.2021.20306)
Supplement: Supplement. — eAppendix. Supplementary Methods eReferences [file jamanetwopen-e2120306-s001.pdf]

Supplemental Online Content

Tian P, Qiu R, Wang M, et al. Prevalence, causes, and health care burden of pleural effusions among hospitalized adults in China. *JAMA Netw Open.* 2021;4(8):e2120306. doi:10.1001/jamanetworkopen.2021.20306

eAppendix. Supplementary Methods
eReferences

This supplemental material has been provided by the authors to give readers additional information about their work.

© 2021 Tian P et al. *JAMA Network Open.*

eAppendix. Supplementary Methods

Patient identification

From the 2018 China Health Statistics Yearbook, we obtained population data and information from hospitals in six regions of China (Northeast, North, East, Northwest, Southwest, and South-Central). All tertiary hospitals in each region were sampled. The ratio of included hospitals in each region's capital and noncapital cities was 1:1. Discharge records of adults 18 years or older who had received a diagnosis of PE and were admitted to the inpatient departments between January 1 and December 31, 2018, were reviewed. Patients with PE confirmed by ultrasound or CT were included.

We set up one panel expressly for this study at the leading study center, West China Hospital (WCH) of Sichuan University. The panel was constituted by 10 authoritative WCH specialists, one in each of the following fields: chest diseases, infectious diseases, cardiology, rheumatology, nephrology, geriatrics, gynecology, oncology, pathology and imageology. Each expert had worked full-time in clinic for more than 20 years and had not retired. The selection of specialists was based on voting by all clinicians in their respective departments.

During the whole study, all the record information of the included cases was gathered and preliminarily classified as cases with/without definite pathogenic or pathological diagnosis. Those without definite pathogenic or pathological diagnosis (a total of 3226 cases) were submitted to the panel, which met twice a week to discuss about 20 cases each time. During a panel review meeting, the panel first decided the range of diseases by distinguishing whether it was exudate or transudate, according to the Light criteria.[1] Then they determined the causes of the cases through a comprehensive analysis of clinical manifestation, laboratory tests, imaging results and

© 2021 Tian P et al. *JAMA Network Open.*

treatment response. The diagnostic criteria of the causes of PE were in accordance with international guidelines. Empyema and parapneumonic pleural effusion were defined according to the criteria of the American Thoracic Society.[2, 3] Malignancy was defined as positive malignant cells in pleural fluid cytology or pleural biopsy.[4, 5] Tuberculosis was defined as tuberculous infection confirmed by pleural biopsy or positive culture of Mycobacterium tuberculosis in PE.[4] Compound causes were defined as two or more causes of PE in one patient.

The exclusion criteria were as follows: 1) discharge time beyond 2018; 2) hospitalization for less than 24 hours; and 3) PE not confirmed by ultrasound or CT scan. For patients with repeated hospitalizations, we included information on only the first hospitalization in 2018.

Data collection

Data from eligible patients were electronically collected from hospital medical records. Before data collection, researchers from each participating hospital provided the hospital's address, the number of hospital beds on all services and the total number of patients discharged in 2018. Data consisted of demographic information, smoking status, alcohol consumption (based on self-report at hospital admission on an ordinal scale comprising never, occasionally, sometimes, and every day), admission and discharge diagnoses, diagnostic methods (imaging techniques, pleural biopsy, etc.), the causes of PE (as determined by clinical exam or pathologic results of pleural fluid or pleural tissue biopsies), the results of auxiliary examinations, treatment details, outcomes (occupancy of ICU by patients in critical condition), medical costs and the payor (Urban Employee Basic Medical Insurance, Urban Resident Basic Medical Insurance, New Rural Cooperative Medical Scheme, etc.). Data quality

© 2021 Tian P et al. *JAMA Network Open.*

control personnel randomly selected 10% of all the patients and verified the accuracy of the data through telephone follow-up.

Statistical methods

We determined the causes of PE for each sex, age group by decile, and smoking status. Our analysis included all participants for whom the data of interest were available. We did not impute missing values. Categorical variables were expressed as numbers and percentages, and continuous variables were expressed as means and standard deviations or medians and interquartile ranges as appropriate. The chi-square test was used to compare the differences in causes of pleural effusion among patients with different smoking statuses. Data were analyzed with SPSS version 21.0 Statistical Software (SPSS Inc., Chicago, IL, USA). Alpha was set at 0.05, and all tests were two-tailed.

eReferences

1. Light RW, Macgregor MI, Luchsinger PC, Ball WC Jr. Pleural effusions: the diagnostic separation of transudates and exudates. *Ann Intern Med*. 1972;77(4):507–513. doi: 10.7326/0003-4819-77-4-507.

2. Niederman MS, Mandell LA, Anzueto A, et al. Guidelines for the management of adults with community-acquired pneumonia. Diagnosis, assessment of severity,

© 2021 Tian P et al. *JAMA Network Open.*

antimicrobial therapy. *Am J Respir Crit Care Med*. 2001; 163(7):1730-1754. doi: 10.1164/ajrccm.163.7.at1010

3. American Thoracic Society, Infectious Diseases Society of America. Guidelines for the management of adults with hospital-acquired, ventilator-associated, and healthcare-associated pneumonia. *Am J Respir Crit Care Med*. 2005; 171(4):388-416. doi: 10.1164/rccm.200405-644ST

4. Ping Luo, Kaimin Mao, Juanjuan Xu, et al. Metabolic characteristics of large and small extracellular vesicles from pleural effusion reveal biomarker candidates for the diagnosis of tuberculosis and malignancy. *J Extracell Vesicles*. 2020; 9(1). doi: 10.1080/20013078.2020.1790158

5. Mattison LE, Coppage L, Alderman DF, Herlong JO, Sahn SA. Pleural effusions in the medical ICU: prevalence, causes, and clinical implications. *Chest*. 1997;111(4):1018-23. doi: 10.1378/chest.111.4.1018

© 2021 Tian P et al. *JAMA Network Open.*
